# Evaluating the Connectivity of a Protected Areas' Network under the Prism of Global Change: The Efficiency of the European Natura 2000 Network for Four Birds of Prey

**DOI:** 10.1371/journal.pone.0059640

**Published:** 2013-03-19

**Authors:** Antonios D. Mazaris, Alexandra D. Papanikolaou, Morgane Barbet-Massin, Athanasios S. Kallimanis, Frédéric Jiguet, Dirk S. Schmeller, John D. Pantis

**Affiliations:** 1 Department of Ecology, School of Biology, Aristotle University of Thessaloniki, Thessaloniki, Greece; 2 Department of Ecology and Evolutionary Biology, Yale University, New Haven, Connecticut, United States of America; 3 Department of Environmental and Natural Resources Management, University of Ioannina, Agrinio, Greece; 4 Muséum National d'Histoire Naturelle, UMR 7204 MNHN-CNRS-UPMC, Conservation des espèces, Restauration et Suivi des Populations, Paris, France; 5 Helmholtz Center of Environmental Research – UFZ, Department of Nature Conservation, Leipzig, Germany; Cirad, France

## Abstract

Climate and land use changes are major threats to biodiversity. To preserve biodiversity, networks of protected areas have been established worldwide, like the Natura 2000 network across the European Union (EU). Currently, this reserve network consists of more than 26000 sites covering more than 17% of EU terrestrial territory. Its efficiency to mitigate the detrimental effects of land use and climate change remains an open research question. Here, we examined the potential current and future geographical ranges of four birds of prey under scenarios of both land use and climate changes. By using graph theory, we examined how the current Natura 2000 network will perform in regard to the conservation of these species. This approach determines the importance of a site in regard to the total network and its connectivity. We found that sites becoming unsuitable due to climate change are not a random sample of the network, but are less connected and contribute less to the overall connectivity than the average site and thus their loss does not disrupt the full network. Hence, the connectivity of the remaining network changed only slightly from present day conditions. Our findings highlight the need to establish species-specific management plans with flexible conservation strategies ensuring protection under potential future range expansions. *Aquila pomarina* is predicted to disappear from the southern part of its range and to become restricted to northeastern Europe. *Gyps fulvus, Aquila chrysaetos,* and *Neophron percnopterus* are predicted to locally lose some suitable sites; hence, some isolated small populations may become extinct. However, their geographical range and metapopulation structure will remain relatively unaffected throughout Europe. These species would benefit more from an improved habitat quality and management of the existing network of protected areas than from increased connectivity or assisted migration.

## Introduction

Conservation networks have been established based on different targets and criteria [1] and actually represent static networks of a given dimension and a constant area. The selection of eligible sites to be included in such networks is based on the current distributions of species, habitats and/or ecosystems. Still such prioritization processes largely ignore reductions of favorable area for species persistence as a result of land conversion, habitat loss, fragmentation and climate change [2]. These limitations raise a critical question on whether existing networks are efficient in conserving biodiversity targets under land use and climate change, and thus whether these networks could safeguard coherence and resilience to these threats [3], [4]. Land use changes and climate change are considered the basic threats to biodiversity [5]–[7], that could significantly reduce conservation efficiency of networks of protected areas [3], [8], [9] by altering their structure and properties [10], [11]. Range shifts due to climate and land use changes could cause species to move out of protected areas, while local extinction could alter the community composition within protected sites, suggesting that these two basic threats should be taken into consideration when setting conservation priorities [4], [12].

Despite the fundamental importance of understanding the impacts of climate change to the species distributional changes and its significance in the effective design of conservation networks [13], only few studies have assessed both climate and land use changes as an element of existing conservation strategies [3], [4], [8], [9], [12]. These studies considered a variety of taxa and examined whether a species that is currently present in a given site will be able to persist in the same site in the near future. Such results have raised serious concerns on the ability of existing networks to protect biodiversity under global change scenarios. Still, assessments of network efficiency are usually drawn by considering changes in site conditions to conserve species under future scenarios eliminating our ability to evaluate impacts at a network-based scale from a macroscopic perspective.

Based on the current status of conservation networks and considering the complex impacts of climate and land use changes, it becomes clear that some sites will inadequately protect species in the near future. The spatial structure, configuration and complexity of protected sites in a conservation network could be indicative of the possibility for individual dispersion within the network or could facilitate the selection of alternative and favorable routes for groups of organisms [14]. A well designed network that ensures connectivity for a given species could theoretically increase persistence to disturbances and climate change by facilitating flow of individuals and/or colonization between neighboring sites. In that sense, although at some sites climatic conditions will not remain suitable for some species, the network itself might be able to protect such species. Based on that line of thinking, most conservation studies on the effects of climate change, recommended that increasing connectivity will decrease the species extinction risk [15]. In any case, the spatial properties of conservation networks should receive additional attention [16] with more complex approaches requiring evaluating the connectivity along with the spatial properties of conservation networks in light of climate and land use changes at continental scales.

Different species, even phylogenetically closely related, might display different responses to climate change (e.g. [17]). Characteristically, the distribution of a given set of sites that could favor exchange and movement for one group of species may not be able to ensure connectivity for a different group of species with more restricted dispersal abilities [16]. Thus, it is critical to examine how such key sites for connectivity are going to fare under climate and land use change. It is likely that a network that appears as a set of interconnected sites could sufficiently ensure connectivity and protection for a group of species with a given dispersal ability when some of these sites will not provide favorable conditions under changing climate and land use transformations. In contrast, the same network might not safeguard conservation of another species with limited dispersal abilities. These hypotheses raise questions on whether conservation priorities should be species and/or scale-specific. These hypotheses also highlight the need to examine whether the efficiency of conservation networks under the risk of climate and land use changes should be addressed considering the loss of some sites as unfavorable or the overall-interconnected properties of the network.

In the present study, we evaluate the overall efficiency of conservation networks in protecting biodiversity by combining two different approaches: species distribution models and graph theory. We developed and tested our approach in Natura 2000, the European Network of protected sites using four raptor species as models. We questioned whether site-based network assessments could adequately predict efficiency of the network to conserve a list of species under global change. Here, we investigated the spatial properties of the entire conservation network. We did so from a macroscopic, network-based point-of-view, in order to provide insights on the robustness or fragility of the network as a result of the removal of sites that would become unsuitable for a species. The coherence of the networks and their ability to ensure connectivity and thus species persistence were studied by comparing network topology statistics.

## Materials and Methods

Our proposed framework included three steps: a) development of species distribution models using climate and habitat variables, b) development of graph models based on modeled current and future distributions of species and c) estimation and comparison of network properties and statistics. We focused our analysis on four species of birds of prey: Griffon vulture (*Gyps fulvus*), Golden eagle (*Aquila chrysaetos*), Egyptian vulture (*Neophron percnopterus*) and Lesser spotted eagle (*Aquila pomarina*). Selected species cover a range of dispersal distances and are widely distributed within the study region allowing to test the applicability of our approach across various scales.

### Species Distribution Models

We initially developed a series of models to predict both present species distribution under current climate and land use, and future species distribution under scenarios of climate and land use change. We used the species distribution modelling techniques as in Barbet-Massin et al. [18] relating presence/absence data to climate and land use variables across the species’ distribution at a 0.5° spatial resolution (∼50 km). The potential future distributions were then obtained by projecting the models under different climate and land use scenarios (Text S1). It should be noted that these techniques do not account for potentially important species interactions but focus on where there will be available suitable habitat for the species [19]–[21], thus it remains unspecified whether the outputs of the models are affected by these plausible interactions. Current and future modeled distributions were overlaid with the Natura 2000 network map available from the European Environmental Agency [22]. The Natura 2000 network map consisted of the boundaries of the protected sites that are distributed across member states of the European Union and is accompanied by information on species presence within each of these sites.

The Natura 2000 conservation network currently consists of more than 26000 sites distributed in all member states of the European Union [23]. For each site, data on species presence were available through the Natura 2000 database [22]. Our model predicts where the species could potentially exist, and thus model predictions could overestimate current and potential future distributions [4], [20], which could provide a source of bias by estimating potential losses of species from areas they do not currently inhabit. Therefore, we maintained only those Natura 2000 sites for which both model predictions and field observations (based on the reporting database of EU on the conservation status of habitats and species according to article 17 of the Habitats Directive 92/43/EEC [24]) coincided. For all four species, there was acceptable coincidence between model predictions and field observations. Less than 15% of the sites with observed presences were classified as outside the species presence according to the model predictions. Possible explanations for these sites might include the presence of species in unsuitable habitat due to ecological mechanisms like extinction debt or source-sink metapopulation structures, or the presence but not the breeding of the species. Similarly, in order to assess network properties based on climate change and land use changes, we overlaid predicted future species distributions to existing protected sites.

### Graph Models and Network Analysis

A graph-theory approach was used to develop network models of potential connectivity among sites based on modeled current and future distributions of the selected species. In graph-theory a graph is composed of two basic elements: nodes (i.e. locations or spatial elements within the network) and edges (which represent the potential linkages between nodes). Here, the centroid of each Natura 2000 site was used as a node of the network. Euclidean distances between the centroids of each pair of nodes were estimated for all sites supporting the presence of a given species. A pair of sites (i.e. pair of nodes) of the network was considered as connected, i.e. to share an edge, if the distance between the nodes is inferior or equal to the estimated dispersal ability of the species [25]. For a given species, dispersal distance was derived using models proposed earlier [26]. These models were based on negative exponential distribution and predict dispersal distances of species considering their body mass and diet types. The four selected species differed in terms of their median dispersal distance (*G. fulvus*: 145 km, *A. chrysaetos*: 90 km, *N. percnopterus*: 54 km, *A. pomarina*: 44 km).

In order to test whether differences in predicted current and future distributions would result in changes in the structure and properties of the networks of interconnected sites, we performed pairwise comparisons of a series of well-defined network topology metrics. The number of edges per node is a simple measure of network topology called node degree, and indicates how many sites interact together. Different networks have different degree distributions, which reflect basic spatial properties and are indicative of network robustness [27]. A scale-free network displays a power-law node degree distribution with several nodes having only few edges and a limited number of high-degree nodes. A well-known property of the highly heterogeneous scale free networks is that they are robust to random removal of nodes but sensitive to the removal of high-degree nodes [28]. In contrast, random networks are less heterogeneous, characterized by a Poisson degree distribution. The robustness of these networks depends on their homogeneity, thanks to which network connectivity remains unaffected by node removal, either random or targeted to higher degree nodes [29].

To examine network topology and combine basic features of network properties for predicted current and future distributions of species we calculated and evaluated a series of network topology metrics, namely number and order of components, articulation points, betweenness centrality and clustering coefficient.

Components are groups of nodes that are connected to each other but have no linkage with nodes outside the component. From a conservation perspective, when a species distribution has several components, this implies distinct metapopulations, which could result in genetic divergence [30] or reproductive isolation on a local scale (unpublished data). Different components could favor species persistence by preventing disease spread but could also prevent individual exchange or colonization after catastrophes. The order of a component is defined as the number of nodes included in it. In a network it is possible to identify nodes whose removal would increase the number of components by separating an existing component into two new ones. These nodes are known as cut nodes or articulation points and are crucial for maintaining network connectivity. Betweenness centrality of a node is estimated as the proportion of the shortest path between every pair of nodes that passes through the given node. Higher values of betweenness centrality are indicative of the higher contribution of a node to large scale connectivity of the network [31]. The clustering coefficient is a measure of network cohesiveness, ranging from 0 to 1 and representing the average fraction of a node’s neighbors that are also directly linked to each other. Higher values of this metric are indicative of a higher probability for an animal in a site (node) to easily move to other sites (nodes) [30].

In addition to the classical network topology metrics, we employed two recently proposed metrics that have been applied for assessing ecological or landscape connectivity with a node, representing a habitat patch, considered as an area where connectivity occurs [32]. These metrics take into account the available habitat area but also the connections among and inside habitat patches as critical factors contributing to dispersal among resource patches [32].

The node-based metric Generalized Betweenness Centrality GBC has been proposed as a more ecologically-relevant version of the classical Betweenness Centrality metric) [33]. This improved index does not simply consider the number of shortest paths that pass through a given node, but also takes into account the area of the nodes that are connected though a given node and the topological distances between the nodes. We further employed the metric Equivalent Connected Area (ECA) that could be defined as the actual size of a single habitat patch that would provide a similar probability value of connectivity than the actual habitat pattern in the studied landscape [34]. The ECA as an improved network based measure of connectivity, which has area units, takes into account three components, (i) connected area that exists within habitat patches, (ii) a dispersal flux between different patches in the landscape and (iii) the importance of patches and edges as connecting elements that support the connectivity between other habitat areas (for more details on algorithms and properties please check [34]–[36]). ECA value will be equal to the total habitat area existing in the landscape when all the habitats have a maximum connectivity and thus are enclosed in a single habitat patch. Following Saura et al. [35], a practical way to assess changes in network connectivity as a result of different undergoing processes, in our case global change, consists in the comparison between changes in the relative variation of ECA (dECA) and in difference in available habitat area (dA). The structure of our networks by default would lead to a reduction in the number of nodes in future networks, and thus a negative value of dA. A negative value of dECA is indicative of a relative decrease in connectivity, which could reflect the loss of some of the initial nodes. A comparison between dA and dECA provides a qualitative way to assess whether the magnitude of connectivity changes is relevant to that of available habitat, with the case of dECA>dA being indicative of a comparatively weaker impact of future changes to connectivity than would be expected by the changes in the habitat area.

We calculated GBC and ECA indices, for every studied species, by estimating their suitable area within each protected site according to their species distribution models. The edge-to-edge Euclidean distances between each pair of habitat patches were then measured. The analyses were performed by using the version 2.6 of the Conefor Sensinode software package [37].

### Statistical Analysis

We used Spearman’s correlation coefficient to investigate whether there is any relationship between the number of nodes that are lost under future changes and the order of their components. To examine whether there is a significant difference in degree, betweenness centrality and GBC between the nodes that are lost due to future changes and those that are maintained in the networks, we used a randomization process that allowed us to extract equal samples of nodes from the networks, obtaining the level of significance after 999 permutations. The same permutation process was repeated to compare the degree of nodes between large and small components (<10 nodes). We further used pairwise Mann-Whitney U-tests to examine potential differences between the clustering coefficient of the networks of the same species based on potential current and future distributions.

## Results

### Network Properties

**Figure 1:**
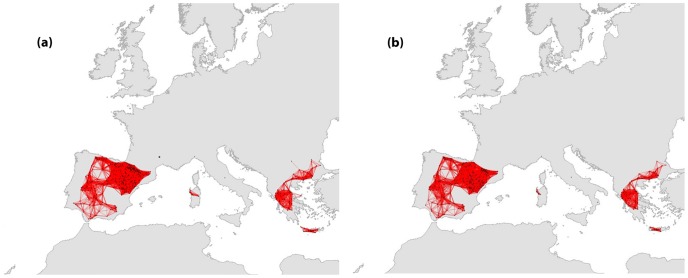
Network structure of the current and future modeled distributions of *Gyps fulvus.*

**Figure 2:**
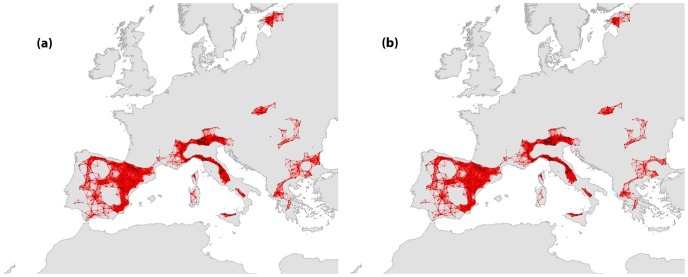
Network structure of the current and future modeled distributions of *Aquila chrysaetos.*

**Figure 3:**
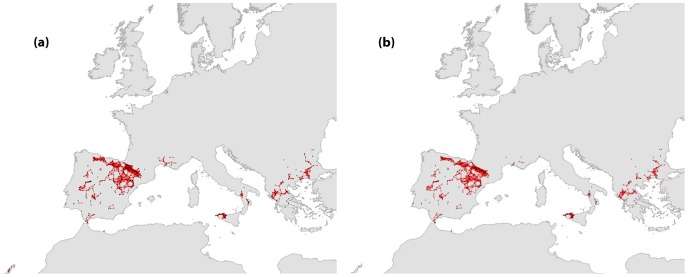
Network structure of the current and future modeled distributions of *Neophron percnopterus*.

**Figure 4:**
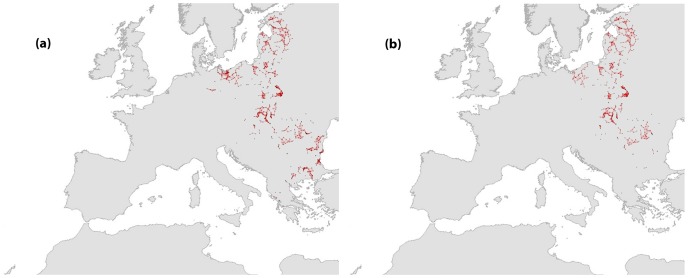
Network structure of the current and future modeled distributions of *Aquila pomarina*.

Based on the current species distribution within the Natura 2000 sites we identified several components for each species (Figure 1, 2, 3, 4, Table 1). The number of components increased as the dispersal distance decreased. Degree and betweenness centrality were significantly smaller for nodes belonging to smaller components (for all species: p<0.01). We identified a statistically significant difference (Mann-Whitney U = 1326, p<0.05) between the order of the components that lost nodes under future conditions (large components Mean = 51.29±165) and those that remained unchanged (small components Mean = 8.21±14.7). Degree and betweenness centrality of nodes that are lost due to climate and land use changes were significantly lower than mean values of the nodes maintained in the networks (in both cases for all species: p<0.01) as were the values of the GBC index (for all species: p<0.01).

**Table 1:** Basic characteristics of the networks (current and future) of the four species.

Species	Median dispersal distance (km)	Number of nodes				Number ofcomponents		Number of isolated nodes		Number of nodes/components		Order of largest or smallest component		Number of articulation points	
		current	future	losses	Percentagelost (%)	current	future	current	future	current	future	current	future	current	future
*Gyps fulvus*	145	522	445	77	14,75	8	7	4	3	65.25±141.2	63.57±126.2	408/1	343/1	0	0
*Aquila chrysaetos*	90	1383	1348	35	2,53	16	14	3	2	86.44±260.69	96.29±276.52	1057/1	1052/1	2	3
*Neophron percnopterus*	54	514	474	40	7,78	35	36	11	14	14.69±43.77	13.17±40.48	257/1	237/1	7	6
*Aquila pomarina*	44	624	465	159	25,48	65	50	18	17	9.6±17.58	9.3±17.35	83/1	83/1	11	12

The degree distribution of each distinct component supporting a significant number of nodes, followed a Poisson distribution indicative of a random network structure (Figure S1). This is actually the case for all four study species. Even after nodes were removed due to unfavorable future conditions at the sites, the networks maintained their structure with the number of nodes decaying exponentially.

The mean clustering coefficient changed slightly as nodes were removed from the networks (for all species p>0.05). For networks constructed based on the current or modeled future distribution of the studied species, mean values of clustering coefficient also demonstrated a gradual decrease for species with lower dispersal abilities (i.e. with values gradually decreasing for networks of networks of *G. fulvus*, *A. chrysaetos*: *N. percnopterus*: and *A. pomarina*), indicative of the high cohesiveness of the networks of large dispersers. The same results were also obtained even after grouping components of the different species based on their order.

Following the loss of nodes due to global changes, the available habitat area was reduced in the predicted future networks (Table 2). The same pattern was observed for ECA which was reduced for three out of the four species we studied, demonstrating changes in network connectivity. However, this was not the case for *A. chrysaetos* with dECA having a positive value indicative of relatively increased network connectivity in the future networks. Still, the decrease in network connectivity was lower than the decrease in habitat area for the other three species (Table 2), suggesting that the loss in terms of area had less detrimental effects on network connectivity than expected.

**Table 2:** Ecological-relevant network based indices for the four species of prey, under modeled current and future distributions; the observed differences in available habitat area (dA) and Equivalent Connected Area (dECA), due to future changes are also presented.

	Equivalent Connected Area (ha)		Total Area (ha)		% change in ECA	% change in total area
	current	Future	current	future		
*Gyps fulvus*	6348903	5592091	13381774	11631070	−11,92	−13,08
*Aquila chrysaetos*	8234347	8282544	25678502	24968535	0,59	−2,76
*Neophron percnopterus*	4044250	3963968	12612170	12223346	−1,99	−3,08
*Aquila pomarina*	4170549	3049496	13959316	9811228	−26,88	−29,72

### Effects of Future Changes on Species Distribution and Connectivity

The distribution of *G. fulvus* covered a large part of southern Europe but extends well beyond Europe to North Africa and Central Asia. In Europe, its distribution in protected areas forms four well structured components ranging from 10 to 482 nodes (129±188.5) and four components consisting of only one node, representing isolated sites (Figure 1). The two largest components were located in the Iberian Peninsula and in the southern part of the Balkans (Greece and Bulgaria), while two medium sized components were found in two Mediterranean islands (Sardinia and Crete). These components appeared to be well connected and as a result no cut nodes were identified. About 14.8% of the nodes (77) of the original network might be lost due to future changes (Table 1). The number of the components was reduced from 8 to 7 under our climate change and land use change scenarios; still the component lost was an isolated site located in the western part of France (Figure 1). The majority of lost sites were located within the large, strongly connected components. In those, we observed a decrease in the mean number of nodes per component but also a reduction of the order of the largest component (Table 3). The network properties of the remaining network differed only slightly from the original one.

**Table 3:** Network Topology for the four species.

	Mean node degreecentrality		Mean betweennesscentrality		Mean clustering coefficient	
	Current	future	current	future	current	future
*Gyps fulvus*	63.16 (±44.12)	50.53 (±34.17)	0.0025 (±0.0045)	0.003 (±0.005)	0,7448	0,7413
*Aquila chrysaetos*	37.86 (±27.04)	38.21 (±27.28)	0.0042 (±0.0161)	0.0045 (±0.0172)	0,7438	0,746
*Neophron percnopterus*	11.44 (±9.79)	10.46 (±7.96)	0.0024 (±0.0043)	0.0026 (±0.0048)	0,7058	0,7079
*Aquila pomarina*	4.85 (±2.93)	4.6 (±2.87)	0.0004 (±0.0008)	0.0008 (±0.0015)	0,6603	0,6466

*A. chrysaetos* was distributed across entire southern Europe as well as in some sites in the northern United Kingdom and in Estonia (Figure 2). Its global distribution covers also sites located in the western part of North Africa (Algeria, Morocco, Tunisia; [38]) but also sites in central and northern Europe. Still, species distribution predicted by models and based on the information available by the Natura 2000 network did not coincide in Central Europe and Scandinavia. For this species, we identified nine large components ranging from 11 to 1057 nodes (142±341), four smaller components of only few nodes (2–3) and three isolated sites (Table 1). A giant component (1057 sites) was identified covering the entire Iberian Peninsula and a large part of the Italian Peninsula extending up to Central Europe. Mediterranean France connects the two large parts of this component, thus representing a cut vertex. In total, two cut nodes could be identified throughout the network. Medium sized components were located in the south of Italy, Sicily, Sardinia, Corsica, Estonia and across the Balkan Peninsula. Our analysis revealed that only 2.5% of the sites (35) might be lost due to future climate and land use changes, reducing the number of components from 16 to 14. The two lost components were an isolated site located in Bulgaria and two sites located in Romania (Figure 2). Most of the nodes were removed from the well connected components, slightly decreasing the order of the largest component. Properties of the future network differed only slightly from the present ones, and in some metrics the score was slightly higher (e.g. mean number of nodes per component) (Table 3).

*N. percnopterus* is also distributed across southern Europe, but its global distribution covers a large part of west and eastern North Africa and South Europe. The European network appeared highly fragmented (Figure 3), with a large component of 257 sites, seven components that supported a significant number of sites (from 11 to 49 nodes), 16 smaller groups of interconnected sites, and 11 isolated nodes (Table 1). The largest component was located at the northern part of the Iberian Peninsula, while medium-sized components were found in other parts of the Iberian Peninsula, in South-Eastern Europe and in southern France. Seven cut nodes were identified in different network components. About 8% of the nodes of the original network might be lost due to future changes (Figure 3). The loss of one of these nodes results in a split of a component into two distinct parts on the Iberian Peninsula. The number of nodes per component and the order of the largest component were reduced in the future network compared to the present one (Table 3).

*A. pomarina*’s global distribution is restricted across Eastern Europe with 12 components consisting of 13 to 83 nodes (40±22.7), 35 smaller components of only a few sites and 18 isolated sites (Figure 4). The largest component covered the Baltic States (Estonia, Latvia and Lithuania) while the other components were scattered across the countries of Eastern and South-Eastern Europe from Poland to Greece. Because of the network layout, several sites could be identified as cut nodes. The rather fragmented structure of the distribution of this species was significantly affected by future changes. More than 25% of the original nodes were removed (Table 1). A total of 15 components were lost indicating local extinctions in all southern populations. Still, given that the southern part of species distribution was lost, the remaining north part of the distribution includes the largest component, which remained unaffected. We obtained only a small reduction in mean number of nodes per components as a result of the loss of few sites from components located at the center of the European distribution of the species (Figure 4).

## Discussion

Here, we developed a graph based approach based on modeled present and future species distributions, in order to test the overall efficiency of conservation networks to protect four bird species under scenarios of climate and land use change. We investigated the spatial properties of the entire conservation network of Europe (Natura 2000). Our analysis demonstrated that the four bird species of our study maintained the network properties of the spatial configuration with well-defined components (i.e. large interconnected sub-networks) despite the loss of nodes (i.e. sites). Different numbers and percentage of sites were excluded as being unfavorable under future climate and land use scenarios. Still, although a number of nodes were omitted, our results showed that the general network structure, defined by node, largest components and network statistics remained unaffected, while the resulting changes in the available habitat area had a smaller impact on connectivity than would be expected by the magnitude of their variation. Our study showed that despite the loss of sites, the spatial distribution of the Natura 2000 network could facilitate connectivity between sites for four birds of prey providing rather robust sub-networks that could support species conservation.

### Global Changes and Network Efficiency

Our analysis has shown that each species was affected by future changes in different ways. Our results offer support to an earlier finding [39] that the concept of umbrella species is not performing well for the preservation of connectivity. The ability of long-distance dispersers (e.g. *G. fulvus*, *A. chrysaetos*) to travel over large areas favors the formation of a few strongly connected clusters with many alternative pathways and only a few smaller components/sub-networks. For such species, any individual located at a given site might be able to cross the giant network components, although the large order of the components increased the number of steps required to travel the entire network. In contrast, species with lower dispersal abilities (e.g. *N. percnopterus*, *A. pomarina*) formed a large number of small components, many of which consisted of only a single Natura 2000 site. In terms of the network analysis, such species needed only a few ‘steps’ to travel among the rather limited number of connected sites but could not benefit from the whole network since the majority of suitable sites were unreachable. For larger distance dispersers the interconnected properties and the numerous alternative pathways (the ability of a given site to be directly connected with many other suitable sites) indicate that this network is resilient to random node removal due to a local catastrophe [40].

Our results indicated that climate and land use changes are not a random process of node removal, but they can be expected to remove sites which are less connected than randomly expected and less critical as dispersal pathways. The protected areas that became unsuitable (i.e. the nodes removed from the network) were not a random sample of areas, but were characterized by a number of features. These sites had less than average edges to other sites and low values of network centrality metrics including the improved modified ecologically relevant centrality measure. Therefore, their removal causes less disruption to the network flow than random node removal. This means that, from a network perspective, even if a number of sites could not retain climate and habitat suitability for a species, the spatial structure of the network would allow a number of alternative sites to be used as potential refuges or stepping stones minimizing the impacts of changes. From a conservation point of view, these findings suggest that for long distance dispersers site-based management policies may prove inefficient, since the movement of individuals is not limited to only few neighboring local populations, which might be the focus of site-specific conservation efforts. It therefore becomes apparent that, for this group of species conservation policy should move from site-specific management to species specific approaches. Still, for the small distance dispersers, (i.e. *A. pomarina*), future changes seemed to result in the loss of several weakly connected components located at the edge of the European distribution which are likely to be ecologically and evolutionary significant units [41], [42] and hence of importance for the survival of the species.

Furthermore, the complex structure of the studied networks, with several components distributed across Europe, illustrated that some of these interconnected groups of sites remained virtually unaffected by climate and land use change. The ecological network-based indices demonstrated a slight reduction in three out of the four studied species, but still these changes were lower than those that would be expected from the variations in available habitat area alone. Cut nodes were not affected by future changes and thus networks retained critical points that could ensure network connectivity. Future changes affected mainly nodes of the largest components but also isolated sites; thus slightly affecting the overall network efficiency. In the case of poor dispersers, the isolation of the multiple small sized components raised some serious concerns on the flexibility of that structure to ensure population persistence, by eliminating the ability of species to disperse and colonize new areas. Even if we assume that climate change would have a greater negative impact on sites located at the southern edge of species distribution, components of small order, even when located at the core of species geographical range, would be expected to have a much higher conservation impact for species with limited dispersal. This is supported by the changes observed in the network structure of *A. pomarina* for which several local rather isolated populations would become extinct. For these species, the increase in available area of a site as well as the inclusion of new sites, which could serve as stepping stones, could increase connectivity and communication between components [43].

Our approach demonstrated that a number of sites (ranging from 4 to 25% of their present day distribution) will be affected and become unsuitable for our model species. *A. pomarina* is mainly affected by climate change, losing all its southern populations. However, the network properties of the remaining northern sites did not change appreciably. Similarly, the comparison of the network topology for the predicted current and future distributions of *G. fulvus*, *A. chrysaetos* and *N. percnopterus* showed little change. Still, for these species the nodes at the southern edge of the species distribution were not affected, but sites were lost in the core of the distributions. It is therefore likely that land use changes, and not climate change (which would affect mostly southern sites), seem to be the most serious threat to their future conservation in Europe. In any case, these findings do not support that climate change would not have a negative effect on global species distribution and persistence of local populations. Our study area was restricted to Europe, with some of the studied species (i.e. *A. chrysaetos* and *N. percnopterus*) having populations at latitudes lower than the southern edge of Europe [38]. These populations (i.e. North-Africa) that are located closer to the equator are more likely to be the first that would face or already have faced the impacts of climate change. In addition, we caution that our conclusions were drawn with respect to four species of birds of prey and any generalization of the findings should be done with caution.

### Limitations and Improvements for Modeling Connectivity of Conservation Networks

The results of our study reflect potential responses of the selected species to global changes. Here, we selected four species with a rather broad distribution, to test and present the applicability of our approach across large scales. Although selected species do actually cover a range of dispersal distances, it is likely that other less mobile species would be much more affected by climate warming and the potential lack or disruption of connectivity among protected areas. We therefore suggest that, similar analysis should be repeated for priority species, which might also have significantly lower distributions or dispersal abilities and thus be even more sensitive to the landscape matrix resistance.

It could further be argued that species respond differently to climate change. Life history traits could affect how species perceive the landscape, while potential spatial constrains (minimum area requirements, carrying capacity etc) even within favorable sites might pose limitations to what could be considered as movement between spatially interconnected sites [39], [44]. Actually, the geographic proximity of two sites could not result in mutual movements and exchange of individuals, if, for example, a barrier (like a large urban center) existed between the sites. In the same sense, although network connectivity has been assessed as a function of distance (e.g. [45] ), inter-patch connectivity patterns could be asymmetric depending on a combination of distance, the direction of movement, and landscape type further affecting metapopulation dynamics and viability [46]. Information on species interactions, community assembly and the heterogeneity of these processes could further improve our knowledge on the sensitivity and effectiveness of conservation. Similarly, more detailed models about the matrix resistance and the linkages with climate and land-use change should ideally allow us to investigate the interactions between landscape features and microevolutionary processes [47], [48]. Still, although fine resolution datasets might now be available, the evaluation of such important interactions, especially in the scale of our analysis, would face serious challenges due to the assumptions about ecological processes and interactions.

We further acknowledge that changes in local conditions at a site level, such as shifts in the seasonal timing of biological events for a group of organisms and potential food gaps [49], could also affect local population viability. However, in the present study we tried to make the most conservative assumptions on how the network will be affected by climate change. We analyzed only the reduction in species range (i.e. without including any information on the colonization of new sites that are currently lying outside the species range but might become suitable after future changes). Another potential mitigation tool would be the establishment of suitable habitat patches located outside the Natura2000 network of protected areas. Such patches will probably play an important role for the connectivity among sites and the preservation of the species, but since these patches are not protected their long term preservation is uncertain, and they may not be available in the future. Thus, we prefer to be cautious, by assuming that only sites within protected areas will continue to exist in the long term. Therefore our methodological framework provides a rather conservative approximation of node inclusion and network connectivity resulting in a more robust evaluation (and most likely an underestimation) of network connectivity both in the present and in the future. It remains an open question how to deal with the uncertainty regarding the long-term preservation of suitable habitat patches that are not protected. Also for estimating the linkage among sites we used a conservative estimation, the median dispersal distance of the species. For example, we assumed that the Egyptian vulture (*N. percnopterus*) has a dispersal distance of 55 km, despite the documented case of long distance dispersal at 550 km in Spain [50]. Thus, we do not overestimate the potential impact of future changes on network structure, since longer dispersal distances lead to even more highly connected networks and even less changes in the network properties of the potential future distribution. We should also mention, that our analysis considered populations already established in Europe, although there are attempts to re-introduce populations of such species in other locations (e.g. ongoing releases of griffon vulture in French Alps and Auvergne).

Recently, Moilanen [51] discussed the limitations of assessing graph-theoretic connectivity, suggesting that the available area of habitats, habitat quality and connection thresholds should be taken carefully into consideration. To this end, we applied a series of alternative measures that explicitly consider habitat conditions [32]–[36]. These indices, to a large extent, verified and supported the findings of the more classical network topology indices. We further suggest that future studies could benefit from analyzing more detailed data on species dispersal potential and thus could allow drawing and analyzing even more specialized graphs (weighted, directed graphs). Furthermore, thresholds of habitat quality and availability, which could be linked to minimum area requirements, could improve the precision of the outputs.

Graph theory could be used for studying the potential impacts of increasing fragmentation, for modeling and supporting the management of fragmented landscapes. Towards this direction few recent studies applied graph theory to evaluate the potential impacts of habitat fragmentation upon biotic communities providing alternative management recommendations at rather fine scales [52]–[54]. Our proposed methodological framework expands on such previous works but also on those that have been developed at national and continental scales [35], [55] which however did not consider potential species distributional patterns and shifts. Recognition of fragmentation patterns and identification of key sites that could facilitate connectivity for target species could improve conservation efficiency and future planning [43] [56].

### Recommendations

One of the most often cited recommendations for biodiversity management in the face of climate change is increasing the connectivity of conservation networks [15]. Under this framework, some plausible solutions to mitigate the impacts of these basic threats could include the establishment of green corridors, the selection and inclusion of key-sites [57] that could somehow increase network efficiency. However, our results indicate that one solution does not fit all problems. The different species of our study displayed vulnerability to different threats. For *A. pomarina*, that loses the southern edge of its range, increased connectivity between southern populations and more northern sites would be profitable. However, for the European populations of *G. fulvus*, *A. chrysaetos* and *N. percnopterus* connectivity is not in danger and, thus, increasing connectivity is not critical. Instead, these species are more threatened by habitat loss, and their conservation demands the preservation of habitat quality. For such species, adaptation and mitigation strategies should focus on the preservation and expansion of protected areas that could reduce the threat of land use changes [58], [59] and guarantee the long-term persistence of local populations.

Considering that a series of studies have demonstrated that land use changes and habitat loss are evident even within protected areas [60], [61], our results provide further evidence towards the need for effective protection at a landscape level. Our findings highlight that there is a need to adapt more species specific conservation policies, and that network based approaches could be used towards the selection of the most efficient measures for each species present in the Natura 2000 network. The framework presented here could be easily applied for assessing the spatial properties of conservation networks and their resilience to future changes. A further development of this methodology may consist of the incorporation of detailed information and traits for different species, such as minimum area requirements and habitat speciation, but also real observations on dispersal distances. In addition, although umbrella species do not apply for connectivity conservation, surrogates such as functional groups of species that share similar behavioral and ecological attributes could ensure a time efficient and comprehensive assessment of the connectivity of protected area networks, towards surrogate-based management recommendations. A network based evaluation of conservation efficiency could further benefit from incorporating elements on the network structure (e.g. node degree heterogeneity) on the spatial ecological dynamics and species life history traits under a metapopulation concept [62], [63].

By means of the efficiency of conservation networks, although future species ranges might include new sites that are not protected under a conservation network, extinctions that could be obtained at the southern areas might not be balanced by potential colonization of such sites. It is therefore critical for efficient conservation under the prism of global change, that a flexible design of the networks should ensure the addition of new sites but also the expansion of the size of already existing protected areas.

## Supporting Information

Figure S1 **Degree distribution for current and future modeled distribution of the studied species.**
(TIF)

Text S1 **Brief description of the methods used for species distribution models.**
(DOCX)
