# Supplementary figures and images for: Evaluating the Connectivity of a Protected Areas' Network under the Prism of Global Change: The Efficiency of the European Natura 2000 Network for Four Birds of Prey
Source: PLoS One. 2013 Mar 19;8(3):e59640. doi: 10.1371/journal.pone.0059640 (PMC3602368; doi:10.1371/journal.pone.0059640)

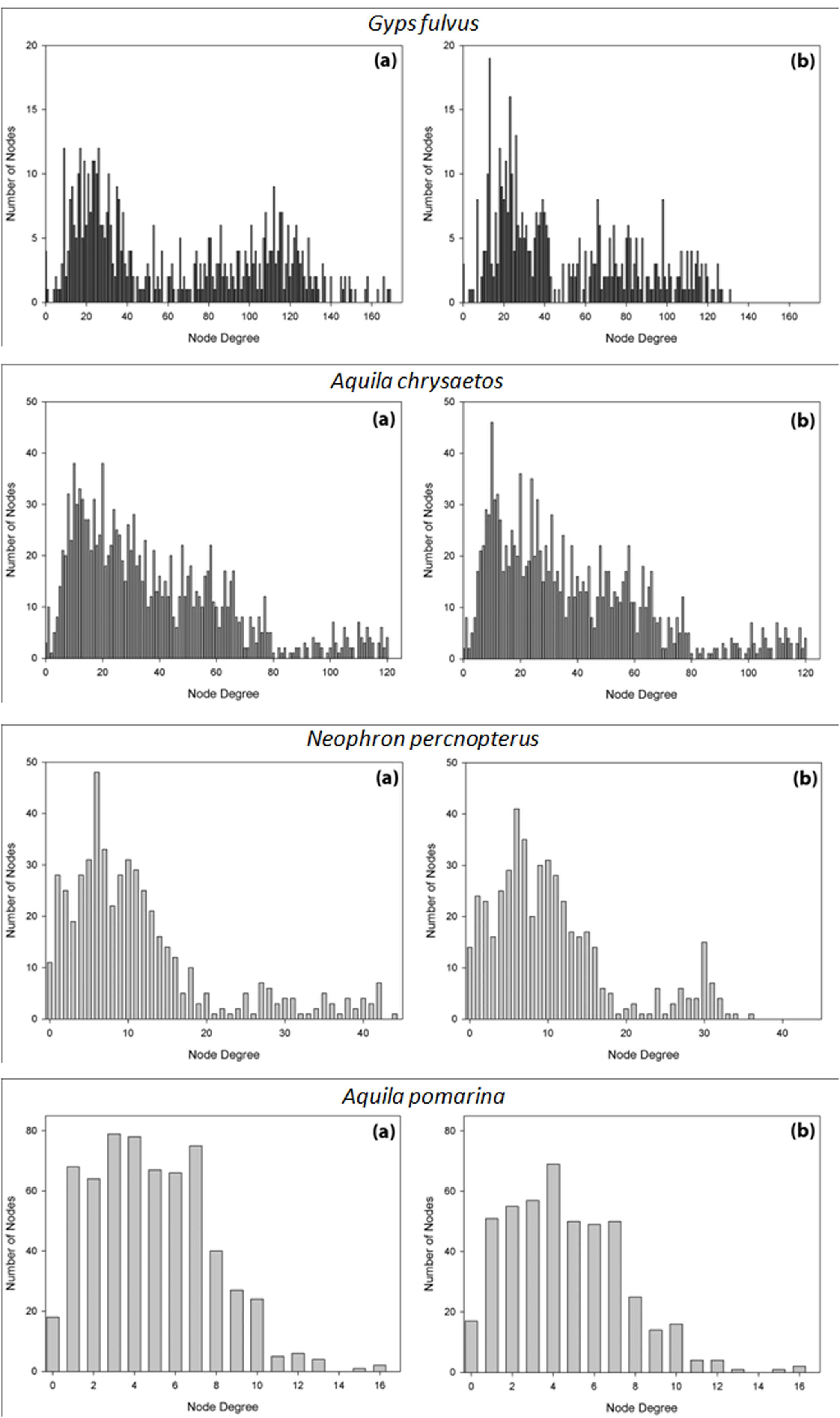

Supplement: Figure S1 — Degree distribution for current and future modeled distribution of the studied species. (TIF) [file pone.0059640.s001.tif]
