# Supplementary material for: Evaluating the Connectivity of a Protected Areas' Network under the Prism of Global Change: The Efficiency of the European Natura 2000 Network for Four Birds of Prey
Source: PLoS One. 2013 Mar 19;8(3):e59640. doi: 10.1371/journal.pone.0059640 (PMC3602368; doi:10.1371/journal.pone.0059640)
Supplement: Text S1 — Brief description of the methods used for species distribution models. (DOCX) [file pone.0059640.s002.docx]

Data:

- **Presence-absence** data were obtained by geo-referencing and digitizing breeding distribution maps. These data were derived from *Birds of the Western Palearctic Interactive (2006) .BirdGuides, Oxford, UK.the handbooks of the birds of the Western Palaearctic. The spatial resolution of presence/absence data was* 0.5°.
- Eight **climatic variables** (mean values over the 1961–1990 period) were derived from Climate Research Unit: (1) annual mean temperature; (2) mean temperature of the warmest month; (3) mean temperature of the coldest month; (4) temperature seasonality; (5) annual precipitation; (6) precipitation of the wettest month; (7) precipitation of the driest month; and (8) precipitation seasonality). The spatial resolution *was* 0.5°.
- 19 land cover types available in the IMAGE 2.4 model (*Integrated modelling of global environmental change (2006). In: An Overview of IMAGE 2.4. (eds Bouwman AF, Kram T, Klein Goldewijk K), pp. 1–228. Netherlands Environmental Assesment Agency (MNP), Bilthoven, The Netherlands*) were used to derive a total of **9 Habitat variables**. These values were averaged over the 1960-1990 period. Data were obtained at a 0.5° resolution.
- **Climate predictions** for 2050 were calculated for three emission scenarios (**SRES**: A1B, B1, and A2, when available) and five calculation models (**GCM**; BCM2, ECHAM5, HADCM3, MIROHIC3_2-HI, and MK3).
- **Future land cover projections** for 2050, were obtained from the three emission scenarios, A1B, A2, and B1, fromthe IMAGE 2.4 model

Niche modeling

- Species distributions were modeled by using a series of methods included within **BIOMOD package** (Thuiller W, Lafourcade B, Engler R, Arau´ jo M (2009) BIOMOD - a platform for ensemble forecasting of species distributions. Ecography, 32, 369–373). These included:
  - three regression methods (**GLM**, **GAM**, and **MARS**),
  - a recursivepartitioning method (**CTA**),
  - three machine-learningmethods (**ANN**, **GBM**, and **RF**)
- A random subset of data (70%) of available information was used to **calibrate** the models.
- A subset of 30% of the data was used to **evaluate the predictive ability** of the models by the means of operating characteristic curve.
- For the final calibration of each model the 100% of all available data was used for **making projections**.
- The consensus acrossdifferent GCM and SRES scenarios was obtained by calculating the mean of the **model obtained from all GCM and SRES**.
- Pixels whose suitability value was above a sensitivity–specificity sum maximization thresholdwere considered as presence.

Detailed description of the methods and the model predictive ability can be found in: Barbet-Massin M, Thuiller W, Jiguet F (2012) The fate of European breeding birds under climate, land-use and dispersal scenarios.Global Change Biology, 18, 3, 881-890
